# Enhancing Emotional Skills of Managers to Support the Return to Work of Cancer Survivors: A Research Opinion Focusing on Value, Feasibility and Challenges

**DOI:** 10.3389/fpsyg.2022.910779

**Published:** 2022-07-12

**Authors:** Marie Viseux, Sietske J. Tamminga, Michiel A. Greidanus, Bertrand Porro, Yves Roquelaure, Marianne Bourdon

**Affiliations:** ^1^UMR INSERM 1246 SPHERE “Methods in Patient-Centered Outcomes and HEalth ResEarch”, Nantes University, University of Tours, Nantes, France; ^2^Amsterdam UMC Location University of Amsterdam, Public and Occupational Health, Amsterdam, Netherlands; ^3^Amsterdam Public Health Research Institute, Societal Participation and Health, Amsterdam, Netherlands; ^4^University of Angers, University of Rennes, Inserm, EHESP, IRSET (Institut de Recherche en Santé, Environnement et Travail) - UMR_S 1085, SFR ICAT, Angers, France; ^5^UMR Inserm S 1085, EHESP, IRSET (Institut de Recherche en Santé, Environnement et Travail) – University of Angers, CHU Angers, University of Rennes, SFR ICAT, Angers, France; ^6^Integrated Center for Oncology, Nantes, France

**Keywords:** emotional skills, return to work, managers, cancer survivors, workplace support, psycho-oncology

## Introduction

The number of new cases of cancer in Europe was estimated at more than 1.6 million for people of working age (20–64 years) in 2020 (International Agency for Research of Cancer, 2020) and ~9 out of 10 cancer survivors return to work 2 years after diagnosis (range 24–94%; Mehnert et al., 2013). Return to work after cancer has often been described as a “return to normal life” (Stergiou-Kita et al., 2014) associated with pleasant emotions (Duijts et al., 2017). However, results from qualitative studies have shown that various emotions may characterize the different stages of the return to work process that are sometimes pleasant, sometimes unpleasant and sometimes experienced together [emotional ambivalence] (Larsen et al., 2001). Cancer survivors may experience this particular state of emotional ambivalence, such as fear together with relief when disclosing their disease state to their manager, or pride to be able to continue working combined with frustration due to reduced cognitive capacities when maintaining work (Robinson et al., 2015; Chapman et al., 2022). Tiedtke et al. (2011) have also found fundamental differences in how cancer survivors perceive their work disability, for example, some will focus on life after treatment, while others feel overwhelmed and exhausted, partly related to intense and alternating emotions. The authors underline the importance of an individual approach to return to work, and support adapted to each experience of work disability. Management support is one of the main needs expressed by the cancer survivors when returning to work, followed by a positive environment and respectful and rewarding communication (Greidanus et al., 2018, 2019). In addition, the intensity and the variability of employee emotions can differ according to the relationship with their manager (Greidanus et al., 2018; Porro et al., 2022). For instance, a strained relationship with the manager before the cancer diagnosis would make returning to work more challenging and vice versa (Stergiou-Kita et al., 2016b; de Rijk et al., 2020).

Managers, i.e., the person directly responsible for overseeing the work, supporting, and coordinating employees within the company, are recognized as relevant stakeholders in the return to work process of cancer survivors (Greidanus et al., 2019, 2020). Therefore, they need knowledge and skills to guide this process and adapt to the needs of cancer survivors as well as their own needs, depending on the specific context of the company (Greidanus et al., 2018; Tamminga et al., 2019). Given the difficult emotional context around cancer for both cancer survivors and managers, supporting the return to work of a cancer survivor is a unique situation for each stakeholder. Stergiou-Kita et al. (2016a) found that misconceptions, fears associating cancer with death and erroneous perspectives on the employability of cancer survivors persist in the workplace. In addition, previous studies reported that managers could experience fear of being burdened, communication problems and, lack of self-confidence or experience (Tiedtke et al., 2012, 2014; McKay et al., 2013). These situations involve a mix of different emotions (Mikolajczak et al., 2009). Managers may have to cope with the above-mentioned employee emotions as well as their own emotions when supporting return to work after cancer (Tiedtke et al., 2017).

To date, managers have tools available in open access web sites or with a web link, targeting managers' actions supporting the RTW process. These tools teach governmental laws, communication models, and return to work planning, relying on videos, conversation tips, checklists, and interactive tools (Greidanus et al., 2020; Maheu et al., 2021). The relevance of focusing on emotional skills should be discussed to complete and broaden the possibilities of developing manager capacities, particularly for consolidating their actions favoring the return to work of cancer survivors, and helping them cope with their challenges in supporting return to work after cancer. In fact, participants in the qualitative study conducted by Robinson et al. (2015) reported that managers could formulate, without realizing it, emotionally unsupportive responses and inappropriate or even frightening comments, negatively influencing the return to work process. They can also be affected by the diagnosis and situations reported by cancer survivor (McKay et al., 2013). This commentary aims to explore the relevance of addressing emotional skills (ES) to help managers when supporting the return to work of cancer survivors. After introducing ES and highlighting the gaps in research for managers' ES development to support the RTW of employees after cancer (1), the benefits of ES interventions and the interest for managers, cancer survivors and companies will be discussed (2), then recommendations and challenges will be addressed (3) before concluding (4).

## Emotional Skills: Definition and Growth

The number and the nature of ES depend and vary according to the approach (cognitive; trait; integrative) and ES models (Mayer and Salovey, 1997; Petrides and Furnham, 2003; Mikolajczak et al., 2009). However, a relative consensus emerges around ES that refer to a set of skills such as identify, understand, express, regulate and use emotions, that are implemented when processing emotional information from one's own emotions and from others' emotions (Mikolajczak et al., 2009; Kotsou et al., 2019; Mikolajczak, 2021). These skills are useful for individuals, as emotions provide information regarding their own needs, are part of the decision-making process and guide their own behavior (Mikolajczak et al., 2009; Mikolajczak, 2021). Authors described three acquisition stages to explain inter- and intra-individual differences regarding ES, namely the first stage of “knowledge”, meaning the person has theoretical knowledge on emotions, the second stage named “abilities”, which is the capacity to implement knowledge and the third stage entitled “dispositions”, assimilated into a trait (Mikolajczak et al., 2009; Mikolajczak, 2021).

Studies have shown that, when applied to the work environment, ES interventions improve the quality of social relationships (Kotsou et al., 2011), decision-making style (Nasef Zaki et al., 2018), management skills (Grant, 2007), teamwork, conflict management (Clarke, 2010), wellbeing (Kotsou et al., 2011), job performance and job satisfaction (Turner and Lloyd-Walker, 2008). ES interventions may also reduce workplace incivility (Kirk et al., 2011). Several of these studies have been included in the systematic review by Kotsou et al. (2019). Based on 46 studies on ES interventions, the authors concluded that ES could develop in adulthood *via* short dedicated programs. The meta-analysis carried out by Mattingly and Kraiger, 2019 on published and unpublished studies addressing ES interventions in adults between 2000 and 2016 has concluded in favor of the same result. In parallel, another meta-analysis on ES interventions showed positive effects on understanding emotions (Mayer et al., 2004) and that positive changes in ES due to these interventions might remain over time (Hodzic et al., 2018). Recently, authors conducted a randomized controlled trial to investigate whether or not an intervention improves the ES of senior executives in a private company. They found lasting and significant improvements in the ability to identify, understand, accept their own emotions and those of others, regulate their own emotions and have a positive attitude toward their employees for those who followed the intervention compared to the control group not exposed to intervention (Gilar-Corbi et al., 2019).

To the best of our knowledge, ES interventions for managers in the context of RTW after cancer have not been studied in the scientific literature, whereas previous research showed that ES interventions could be easily implemented and assessed (Kotsou et al., 2019; Mattingly and Kraiger, 2019). Current research primarily focuses on cancer survivors' ES in the return to work process with one study conducted by Gómez-Molinero and Guil (2020). The descriptive and mediation analysis results suggested that developing breast cancer patients' ES could enhance work ability and probability of successful RTW. Nevertheless, it is not all dependent upon cancer survivors' ES. Managers' ES may also be implicated in the successful RTW of their employees with cancer diagnoses.

## ES and Return to Work Interventions

One of the concerns raised by managers is that they may feel uncomfortable with emotions expressed by cancer survivor (Tiedtke et al., 2017) and do not know how to provide the support that favors successful and sustainable return to work. Second, managers have to cope with the uniqueness of each return to work of cancer survivors and their associated specific needs (Tiedtke et al., 2011, 2017; Porro et al., 2022). Also, they expressed the need to strengthen their abilities to adapt to the situation as well as to identify and promote the expression of the specific needs of cancer survivors in a relationship sometimes charged with emotions for the cancer survivor and their manager (Tiedtke et al., 2017). Third, managers can also cope with dilemmas when supporting the return to work of a cancer survivor. For example, managers may experience difficulties in conciliating their professional role with the imperatives of production and their human role of supporting the cancer survivor, or difficulties in combining the interests of the cancer survivor together with those of colleagues regarding workload (McKay et al., 2013; Tiedtke et al., 2014, 2017). These situations can be stressful for managers and influence their health, working relationships, and by extension, the team.

ES interventions would support managers on these three points. On one hand, they address emotions in interpersonal relationships and communication. On the other hand, they aim to increase cognitive flexibility (“i.e., capacity to switch a behavioral response according to changes in the environment”) to adapt to emotionally complex or stressful situations (Mikolajczak et al., 2009; Kotsou et al., 2011, 2019; Mikolajczak, 2021). Moreover, a systematic review assessing ES of health professionals, who may also have a managerial role, have shown that physicians' ES can positively influence interpersonal communication skills, confidence and satisfaction of patients, and decrease anxiety when occupational stress is experienced (Arora et al., 2010). These results also argue in favor of the relevance of addressing ES in interventions dedicated to managers supporting employees with cancer. Whatever their initial ES, interventions could strengthen their achievements and further develop their ES to gain more comfort and confidence when supporting return to work for cancer survivors.

## Discussion: Recommendations and Challenges

Almost 2 million new cancer diagnoses will be made each year in Europe for people of working age (International Agency for Research of Cancer, 2020). Approximately 24–94% of them will return to work (Mehnert et al., 2013). Therefore, many companies will be concerned in the forthcoming years by the implementation of preventative actions to support cancer survivors and the managers who accompany them in returning to work. The first recommendation would be to intervene on ES development and return to work processes in companies as well as in management schools, to raise awareness among future managers and leaders. The choice to offer a face-to-face, remote or hybrid intervention depends on the objectives of the intervention and the availability of the public concerned (companies or schools). The second recommendation could relate to the adaptability of the intervention. Managers should be given the option to choose the format of the modules on ES theoretical knowledge. Similarly, a face-to-face or hybrid format could be proposed for the ability stage (Mikolajczak et al., 2009; Mattingly and Kraiger, 2019; Mikolajczak, 2021). ES interventions in companies to support the return to work of cancer survivors is an emerging field of interest, which lacks elements on interventions content, research protocols and assessments. The transparent reporting of the interventions is a guarantee to evaluate the validity and efficacy of the interventions. This could facilitate improved understanding of the effects of the ES intervention on managers, difficulties encountered, and positive elements to strengthen to support the return to work process after cancer. Therefore, the third recommendation is to encourage the publication of intervention protocols and use TREND or CONSORT guidelines in study reports (Altman et al., 2001; Des Jarlais et al., 2004).

Several challenges deserve particular attention. First, the representation of a “short format” for an intervention can vary according to the country and/or to the size of the company (Tamminga et al., 2019; Tiedtke et al., 2020) and should be considered while developing ES interventions. Second, to allow the transfer of a new ES following the intervention, the manager must have the time to put it into practice in the professional environment, which may require commitment from the company (Gilar-Corbi et al., 2019; Mikolajczak, 2021). Third, the involvement of managers in the ES intervention can be related to motivation and their expectations (Kotsou et al., 2019). Communication works upstream by presenting the intervention, removing prejudices and promoting the intra and interpersonal benefits. Fourth, the difficulties expressed by managers over ES interventions, such as unfeasibility or lack of realistic tools, should be taken into account for the design of future tools (Thory, 2013). Fifth, cancer survivors' sector of activity (e.g., industry, administration) and employment type (e.g., skilled or unskilled) are important factors in the cancer survivor's decision and return to work timing. For example manual work and stressful jobs discourage survivors from returning to their previous employment (Chow et al., 2014; Islam et al., 2014). Also, the means available in companies to support return to work, particularly, to adapt the work, share the activity, and participate in an ES intervention for managers, are more important in large than small companies (Tiedtke et al., 2020). ES interventions should be adapted to the managers' needs, whatever the company's size, sector and activity. Sixth, the emotional norms concerning the expression and the emotional regulation linked to the company's culture (Tran, 2012) could be a barrier or a facilitator to the ES intervention for some managers. Finally, cultural differences in the professional context can influence the expression of emotions in interpersonal situations and their regulation (Matsumoto et al., 2008). The moderating effect of the characteristics of managers and cancer survivors is a challenge to be taken into consideration during ES intervention evaluation (Kotsou et al., 2019).

## Conclusion

Return to work after cancer is a hot topic. Intervening on ES in this context is a useful proposal for managers who express the need to develop these skills as well as managers who would like to strengthen them. ES are useful at an interpersonal level in the relationship between managers and cancer survivors and useful at an intrapersonal level for managers to gain more comfort and confidence when supporting the return to work of cancer survivors. In addition, ES interventions would make it possible to promote exchanges between peers on their practice or experience of return to work support, which refers back to the notion of “emotional regulation” in the definition of ES. However, this will only be possible if companies and management schools consider return to work support after cancer as a process in which the actions of managers can have an essential relational dimension that favors successful and sustainable return to work.

## Author Contributions

MV conceived the ideas presented in this article and wrote the first draft. MB, ST, MG, BP, and YR contributed with discussion on the ideas presented and edited the manuscript. MB contributed with important intellectual content and supervised the whole process. All authors contributed to the article and approved the submitted version.

## Funding

This manuscript was prepared in the context of the SIRIC ILIAD Program supported by the French National Cancer Institute (INCa), the Ministry of Health and the Institute for Health and Medical Research (Inserm); contract INCa-DGOS-Inserm_12558.

## Conflict of Interest

The authors declare that the research was conducted in the absence of any commercial or financial relationships that could be construed as a potential conflict of interest.

## Publisher's Note

All claims expressed in this article are solely those of the authors and do not necessarily represent those of their affiliated organizations, or those of the publisher, the editors and the reviewers. Any product that may be evaluated in this article, or claim that may be made by its manufacturer, is not guaranteed or endorsed by the publisher.
